# Assessment of a Manual Method versus an Automated, Probability-Based Algorithm to Identify Patients at High Risk for Pharmacogenomic Adverse Drug Outcomes in a University-Based Health Insurance Program

**DOI:** 10.3390/jpm12020161

**Published:** 2022-01-26

**Authors:** Kendra J. Grande, Rachel Dalton, Nicolas A. Moyer, Meghan J. Arwood, Khoa A. Nguyen, Jill Sumfest, Kristine C. Ashcraft, Rhonda M. Cooper-DeHoff

**Affiliations:** 1 Invitae, Denver, CO 80134, USA; kendra.grande@invitae.com; 2 Department of Pharmacotherapy and Translational Research, College of Pharmacy, University of Florida, Gainesville, FL 32610, USA; racheldalton@cop.ufl.edu (R.D.); khoanguyen@cop.ufl.edu (K.A.N.); 3 Invitae, Seattle, WA 98121, USA; nick.moyer@invite.com (N.A.M.); Kristine.ashcraft@invitae.com (K.C.A.); 4 Tabula Rasa Healthcare, Moorestown, NJ 08057, USA; marwood@trhc.com; 5 GatorCare, University of Florida, Gainesville, FL 32610, USA; sumfej@shands.ufl.edu; 6 Division of Cardiology, College of Medicine, University of Florida, Gainesville, FL 32610, USA

**Keywords:** pharmacogenetics, pharmacogenomic testing, Precision medicine, ambulatory care, drug interactions

## Abstract

We compared patient cohorts selected for pharmacogenomic testing using a manual method or automated algorithm in a university-based health insurance network. The medication list was compiled from claims data during 4th quarter 2018. The manual method selected patients by number of medications by the health system’s list of medications for pharmacogenomic testing. The automated method used YouScript’s pharmacogenetic interaction probability (PIP) algorithm to select patients based on the probability that testing would result in detection of one or more clinically significant pharmacogenetic interactions. A total of 6916 patients were included. Patient cohorts selected by each method differed substantially, including size (manual *n* = 218, automated *n* = 286) and overlap (*n* = 41). The automated method was over twice as likely to identify patients where testing may reveal a clinically significant pharmacogenetic interaction than the manual method (62% vs. 29%, *p* < 0.0001). The manual method captured more patients with significant drug–drug or multi-drug interactions (80.3% vs. 40.2%, respectively, *p* < 0.0001), higher average number of significant drug interactions per patient (3.3 vs. 1.1, *p* < 0.0001), and higher average number of unique medications per patient (9.8 vs. 7.4, *p* < 0.0001). It is possible to identify a cohort of patients who would likely benefit from pharmacogenomic testing using manual or automated methods.

## 1. Introduction

Clinical information regarding the utility of pharmacogenomics (PGx) is widely available but the best way to identify patients who would benefit from testing is not well defined [1,2,3,4]. Pharmacogenomic testing can predict medication-related adverse reactions, indicate medication changes that can reduce such reactions, and identify the potential for a poor response or treatment failure from a medication. Pharmacogenetic guidelines for many drug–gene combinations are available from the Clinical Pharmacogenetics Implementation Consortium (CPIC), providing support for the implementation of clinical practice decisions based on pharmacogenomic test results [5,6,7]. As of September 2020, CPIC has published 24 guidelines covering 62 different medications. In addition to CPIC, pharmacogenetic guidance is present in hundreds of U.S. Food and Drug Administration (FDA) approved medication package inserts and in other sources [8,9].

Infrastructure resources and cost constraints may limit the ability of health systems to perform pharmacogenomic testing on all patients [3,10,11,12]. Preemptively identifying those patients who will benefit most from pharmacogenomic testing has been a goal of healthcare institutions and the pharmacogenomics community, and ultimately may allow for the most effective and efficient use of information technology, laboratory, and clinical resources [13,14]. Though preemptive testing currently is performed by some health systems, implementation is inconsistent. A recently published tutorial outlines the many necessary steps required to transition from single gene testing when a medication is being considered to a preemptive panel-based genotyping approach [15].

Methods used previously to identify patients for pharmacogenomic testing include overall medication count, significant drug–drug interaction counts, and various spend measures (e.g., ER visits or total healthcare spend) [3,8,10]. However, it is unknown if these manual methods effectively identify the patient populations that will benefit most from pharmacogenomic testing. Although additional validation is needed, the automated algorithm was tested in a prospective randomized controlled trial of patients at high-risk for readmission, where pharmacogenomic testing and YouScript algorithm information were provided to pharmacists in the intervention arm. The automated algorithm in this readmission study calculated a pharmacogenetic interaction probability (PIP) of 33.2% for the intervention and 34.3% for the control group. The intervention arm showed a reduction in the number of re-hospitalizations, ER visits, and composite number of re-hospitalization + ER visits at 60 days of 52% (*p* = 0.007), 42% (*p* = 0.045) and 48% (*p* = 0.01), respectively. An 85% reduction (*p* = 0.054) in mortality was also observed in the intervention group [16].

A limitation to more widespread implementation of preemptive pharmacogenomic testing is uncertainty over which patients to test. It is now possible to use an algorithm to determine which patients might benefit most from testing. The type and utility of the algorithmic approach is dependent on the infrastructure and informatic tools available at a given institution to create the algorithm, access the relevant data, and return usable results. There are automated algorithms available that can analyze population-level data from an institution or healthcare system, and rank the patients based on the likelihood that pharmacogenomic testing will find clinically significant pharmacogenetic interactions [16,17,18]. Whether an automated method identifies the same or different patients as does a locally developed manual identification method or is more useful in identifying relevant patients than the manual identification method is unknown. Therefore, the objective of this project was to compare two groups of patients identified as most likely to benefit from pharmacogenomic testing by comparing the cohorts found using a locally developed manual algorithm versus a commercially developed automated algorithm in a large university-based health insurance program patient population.

## 2. Materials and Methods

### 2.1. Study Population

Prior to development of the patient cohort, the project was approved by the University of Florida (UF) Health Sebastian Ferrero Office of Clinical Quality & Patient Safety, in conjunction with the UF Institutional Review Board. Additionally, a data use agreement was established between UF Health and YouScript that allowed for the sharing of data between the two entities for the purposes of this study only.

The eligible patients were insured through GatorCare, a commercial employer- sponsored, self-funded medical and pharmacy benefit plan. Included patients were adults ≥18 years of age. Medical claims were extracted from the Blue Cross Blue Shield of Florida and pharmacy claims data were extracted for the project from the Magellan Rx Management System by the GatorCare program. Claims data included patient age, sex, number of ER visits and associated costs, number of inpatient visits and associated costs, and pharmacy claims data including prescription fill date, 11-digit NDCs, and additional prescribing and prescription fill data.

A total of 38,402 patients had prescription claims data between January 2017 and December 2018. To identify patients most likely to be active in the health care system, the patients were filtered to identify those with prescriptions filled in Q4 2018 (*n* = 20,365). Further filtering was performed to exclude patients with no claims for the predefined list of pharmacogenetic recommended medications (*n* = 13,441) and those without complete demographic data (*n* = 8), resulting in an eligible cohort of 6,916 patients. Prescription data was converted from 11-digit NDCs to RxCUI using the publicly available RxNorm API from the National Library of Medicine [19]. NDCs that did not map to an RxCUI were excluded. These primarily encompassed non-drug items such as lancets, test strips, and certain OTCs.

### 2.2. Study Design

The objective of this study was to compare two different methodologies, a locally developed manual method and a commercially developed automated algorithm, to identify the patients most likely to benefit from pharmacogenomic testing. The patients in the eligible cohort of 6916 patients were analyzed by the manual and automated method and subjects identified up to a preset limit of ~250. The preset limit was set based on available funds to provide genotyping. Identical pharmacy and medical claims data were used in both methods. The manual method was conducted by members of the Precision Medicine Program at UF Health in Gainesville, FL. The automated algorithm was completed by YouScript, now Invitae, in Seattle, WA. The study was limited to the eight genes on the UF Health pharmacogenomic test panel (Table 1). Both methods included medications affected by these eight genes but differed in the criteria applied for determining relevant medications for inclusion. The manual method used medications listed in columns C and D while the automated method used medications listed in columns C and E. There are differences in the number of drugs included by each method, the majority of included drugs (*n* = 34) are included in both methods. The manual method includes an additional 9 medications while the automated method included an additional 25 medications. The reason for this difference is that the manual method medications are selected based on strength of evidence as well as utilization of the medications in the clinics vs. the automated method medications drugs which are selected by CPIC A or B evidence or actionable FDA labeling.

### 2.3. Manual Method

The manual method included medications in Table 1 columns C and D. These are medications currently used for pharmacogenetic dosing guidance by the UF Health Precision Medicine Program. This program provides evidence-based pharmacogenetic guidance on drug and dose selection. The manual algorithm selected patients by ranking them based on the number of unique medications on their profile (Table 1, columns C and D) during Q4 2018. Ties were broken using the overall unique medication count. Any remaining ties were broken randomly.

### 2.4. Automated Algorithm

The automated algorithm is a patented clinical decision support tool designed by YouScript [20] that calculates the probability that pharmacogenomic testing will result in the detection of one or more clinically significant pharmacogenetic interactions for that patient (Pharmacogenomic Interaction Probability (PIP) score). The PIP score is similar to other pre-test probability scores used to determine risk, such as the Diabetes Risk Score [21] and has been utilized by payers and healthcare providers in numerous EHR-integrated deployments to determine patients most likely to benefit from pharmacogenomic testing.

The PIP score is calculated using the patient’s current medication list, the prevalence of certain pharmacokinetic and pharmacodynamic phenotypes in the North American population, and potential drug–gene interactions with a moderate or higher interaction level. Although racial and ethnic variation is known to impact prevalence, race is an indistinct construct not accurately recorded in medical records and therefore not incorporated in the PIP score. Moderate interactions are the lowest drug–gene interaction level with clinically actionable recommendations based on current published evidence (i.e., actionable FDA labeling or CPIC level A or B). The medications that met this threshold at the time of the study and thus were included in the automated method are listed in Table 1, columns C and E.

The PIP score also accounts for phenoconversion. Phenoconversion is a phenomenon where an individual’s genotype-predicted phenotype can be converted to another phenotype because of a drug interaction (e.g., conversion of genotypic normal metabolizers or intermediate metabolizers into phenotypic poor metabolizers in the presence of a strong inhibitor), thereby potentially modifying their response to a medication [22]. The prevalence of drug–gene interactions has been estimated at 14.7% and drug–drug–gene interactions at 19.2% in tested patients, highlighting the importance of simultaneously consideration of both drug and gene interaction risk [23,24]. The YouScript PIP score usually includes 14 different high evidence genes. However, as the health system only tests for the eight genes in Table 1, the YouScript algorithm was adjusted to include only those eight genes. This change produced the modified PIP scores used in this study. The modified PIP score was calculated for all patients, and the patients were ranked highest to lowest. Ties were broken using number and severity of drug–drug interactions, then using unique medication count.

Patients were placed into four groupings based on PIP score: (1) high, PIP score greater than 50%, which indicates that an actionable drug–gene or drug–drug–gene interaction is more likely to be detected than not, (2) low, the 1–25% and (3) moderate, the 26–50% groups evenly split patients into less or more than 1 in 4 patients needed to test (NNT) to find something actionable, respectively, and (4) none, the PIP score is 0%. A recent publication using the same PIP groupings showed an increase in length of stay for hospitalized Medicare COVID 19 patients in higher PIP risk groups. Interestingly, Medicare risk adjustment factor (RAF), a measure of health status, did not correlate [25].

A randomized controlled study showed that the PIP score for tested patients aligned with gene interactions detected [17]. Descriptive Invitae internal data of over 30,000 patients with medication regimen information provided at time of testing also supports PIP score alignment with the number of substantial gene interactions found.

In addition to the PIP score, YouScript’s interaction algorithm calculated the count and severity level (i.e., contraindicated, severe, moderate, minor, or minimal) of drug–drug and multi-drug interactions for all patients. These counts did not include gene-related interactions, as those interactions are not known until pharmacogenomic test results are complete.

## 3. Results

**Figure 1:**
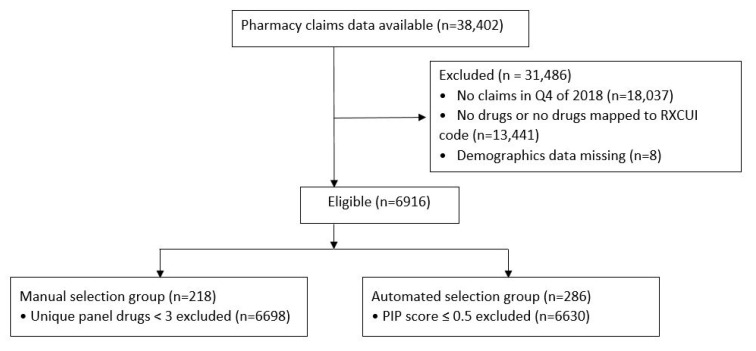
Flow diagram of patients with pharmacy claims included in the study.

The overall flow of patient and prescription claims data in the study is summarized in Figure 1.

Patient demographics data are summarized in Table 2. Patients in the eligible cohort were relatively young with an average age of 35 years and there was no significant difference in age compared to the patients selected by the manual or automated method. Overall, more females than males were included in both cohorts, in accordance with the distribution of the eligible population.

### 3.1. Manual Method Results

The manual method generated a cohort of 218 patients, which was the selection point closest to the 250-patient goal. The 218 patients had claims for three or more medications in the list. Using a cutoff of two or more medications identified a total of 1128 patients, which exceeded the target of 250.

### 3.2. Automated Algorithm Results

The automated algorithm generated a cohort of 286 patients based on the inclusion of patients with the highest PIP scores of 51% and above. A cohort size of 286 was the natural selection point closest to the 250-patient goal in the PIP score results. As the PIP score is a whole-number percentage, multiple patients may have the same PIP score based on medication lists with similar probabilities. Patients in the selected cohort (*n* = 286) had a mean PIP score of 61.6% with a range of 51–81%.

### 3.3. Comparison of Cohorts

**Figure 2:**
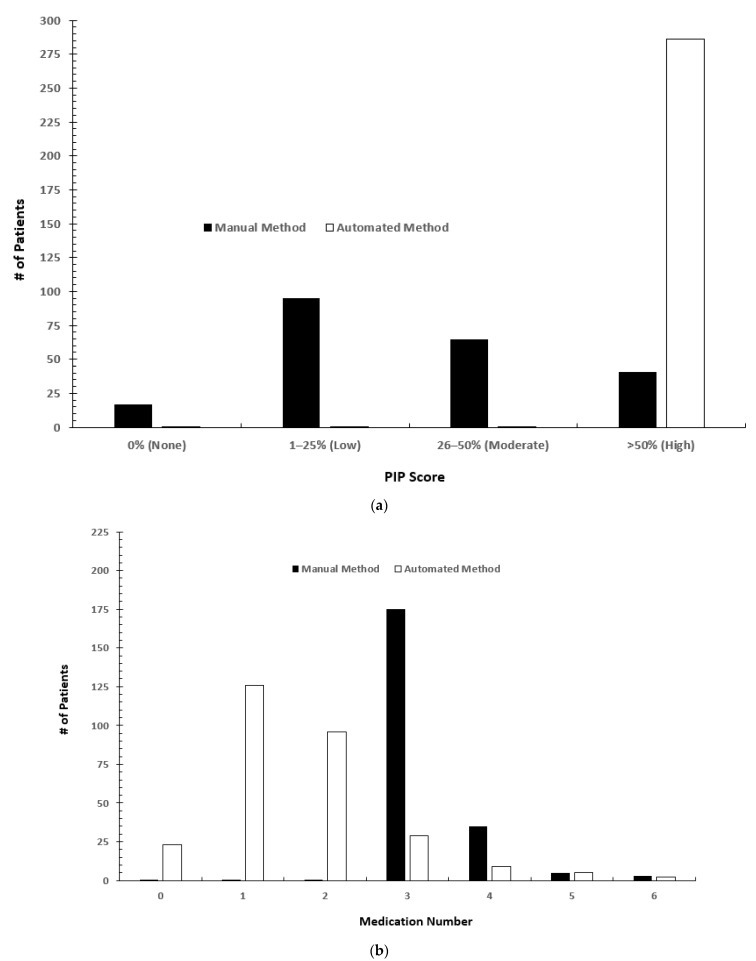
(**a**) Comparison of automated pharmacogenetic interaction probability (PIP) in two cohorts. (**b**) Comparison of manual PGx impacted medication count in two cohorts.

Forty-one (19%) of patients were selected by both methods. These patients had both medication claims for three or more medications and PIP scores in the high (>50%) range. Figure 2a shows the PIP breakdown for patients from both methods. The majority of the manual cohort had PIP scores in the low (1–25%) range (*n* = 95) followed by the moderate (26–50%) range (*n* = 65). All automated method patients were in the high (>50%) range (*n* = 286). Figure 2b shows the number of patients taking a medication included in the manual medication list (Table 1, D. Manual Only). Most of the patients in the automated cohort had claims for at least one medication on the medication list used in the manual method (*n* = 263, 92%). A total of 126 (47%) patients had claims for one medication and 96 (36%) had claims for two medications.

Average unique medication counts were higher in the manual cohort (9.8 vs. 7.4, *p* < 0.0001) (Table 3). Compared to patients identified by the manual method, the patients identified by the automated method had significantly lower average drug interaction counts (3.3 vs. 1.1, *p* < 0.0001) and a lower proportion of patients with at least one moderate-or-higher severity drug interaction (80.3% vs. 40.2%, *p* < 0.0001). The manual method also selected significantly higher average unique medication counts compared to the automated method (9.8 vs. 7.4, *p* < 0.0001). Compared to the automated method, the manual method selected more patients with ER (26.6% vs. 11.2%, *p* < 0.0001) or inpatient visits (19.3% vs. 6.6%, *p* < 0.0001). The average cost was significantly higher for ER visits (USD 558 vs. USD 253, *p* = 0.001) in the manual method but not for inpatient visits (USD 4,423 vs. USD 3,607, *p* = 0.68) (Table 3).

### 3.4. Medication Usage

**Figure 3:**
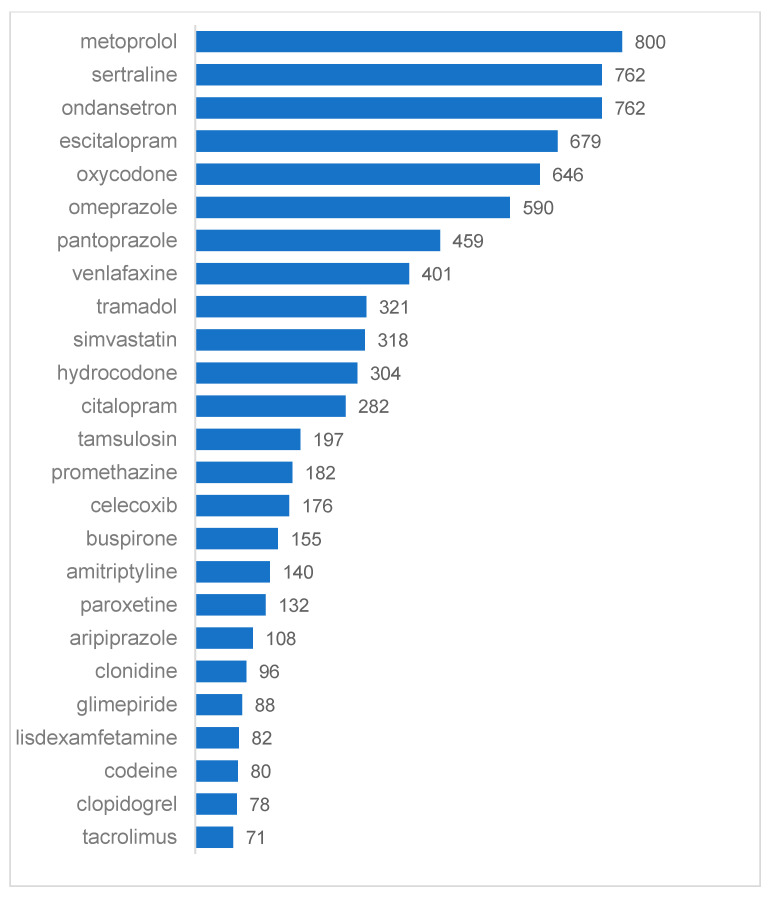
Medications claims count in the eligible cohort (*n* = 6916) in Q4 2018.

In descending order, Figure 3 lists the total number of patients with a claim for an included medication in Q4 2018. Metoprolol, ondansetron, and sertraline were the most frequently prescribed medications. A total of 73.3% of patients had at least one pharmacy claim for at least one unique, included medication. A majority of patients (57%) had claims for one, 13.1% had claims for two, 2.5% had claims for three, and fewer than 1% had claims for four or more unique, included medications.

## 4. Discussion

This study demonstrated the differences between a manual and an automated method to select patients for pharmacogenomic testing in a university-based healthcare system. The differences found between our two methods are important references for clinical informatics consideration, especially when implementing preemptive pharmacogenomic testing programs. To our knowledge, this is the first study to compare these two approaches.

The case has been made for preemptive pharmacogenomic testing for all patients as greater than 99% of patients have been found to have one or more actionable pharmacogenetic variants [26,27]. A recent publication of almost half a million patients in the UK biobank project showed that on average, patients had an atypical response to over 12 drugs with CPIC guidance. This has the potential to impact one in 11 new prescriptions [24]. Even if preemptive testing is provided to all patients, operational or budgetary constraints may require some method for selecting patients most likely to benefit so limited resources can be allocated.

The goal for both methods in this project was to identify patients for pharmacogenomic testing who would benefit from genotype-guided medication management. The primary characteristics considered in each method were very different from each other. Unsurprisingly, the resulting chosen patient cohorts were significantly different, with minimal overlap. In fact, among the 218 patients identified by the manual method and 286 by the automated method, only 41 were identified by both methods. Other methods previously used to select patients for pharmacogenomic testing have included overall medication count, significant drug interaction counts, and various spend measures (e.g., ER visits or total healthcare spend) [8,9,10,11]. Our project worked to improve upon these previously used techniques by selection methodologies specifically focused on current medications where pharmacogenomics may provide clinical improvements as well as targeting the genes available at a specific site.

Both methods targeted patients where pharmacogenomic testing may result in clinically actionable recommendations based on the patient’s current medications (e.g., FDA labeling or CPIC level A or B). This was the primary approach for the automated method and the number of medications included by the automated approach was much larger than the manual approach. While the number of medications from the manual cohort list, taken by the two cohorts, differed considerably they appear higher in the manual method (Figure 2b). This is likely because the automated method included a much longer list of medications than are accounted for in the manual count subset. Additionally, the manual method treats all medications equally so would default to the highest number whereas the automated method weights medications according to the likelihood that testing will reveal an actionable gene-based interaction which varies by gene, what phenotypes have interactions, and whether or not the patient is also taking an inhibitor or inducer. In addition, this method considered both the population phenotype frequency and clinical actionability of the drug–gene interactions into account, as well as including a larger range of medications. Expanding the selection criteria to include these additional elements likely accounts for the resulting patient identification with higher PIP scores. Given the complexities related to phenotype probabilities, it would be difficult to include these elements in a manual method.

While the manual method correlated with higher rates of healthcare utilization, this may be attributable to the higher medication count for that group. As the cost data came from the same quarter (Q4 2018) as the medications, it is not surprising that patients with more healthcare spending had higher medication counts. It is likely that the healthcare event increased the medication count instead of vice versa. Recently discharged patients often have more medications [28]. As expected, we did not see as strong of a cost correlation with the automated method as PIP score is less strongly correlated to medication count.

Results may have been affected by the different medications included in each method. Most medications with significant usage were used in both approaches with the exception of warfarin and metoprolol. The automated method does not include warfarin because dosing for patients already started on warfarin is commonly managed by a blood test that reflects therapeutic levels, rather than management by pharmacogenomic results, unless the patient is unstable. The primary benefit is typically seen in patients who complete pharmacogenomic testing before they begin warfarin [29]. Thus, warfarin is not included in the PIP score. This allows healthcare systems to focus limited testing dollars on other patients who may benefit more. The manual method does not include metoprolol as there was no CPIC published guideline at the time the project was ongoing. The automated method does include metoprolol as there is a significant drug exposure change according to the FDA product insert which states that “poor CYP2D6 metabolizers exhibit several-fold higher plasma concentration of [metoprolol] than extensive metabolizers” [30]. Other publications have shown that, to realize the full benefit of pharmacogenomic testing, identification of testing candidates should be automated inside each health system’s EHR, providing guidance when patients are testing candidates and alerting the clinician when there are relevant drug–gene interactions after testing. As prescription medication lists change regularly, a static pharmacogenomic lab report is quickly outdated and does not provide the benefits shown by real-time alerting [1]. A study comparing use of a bioinformatic tool to a clinically established counseling process for medication reviews including pharmacogenomic testing, found that the tool reduced patient review time from 3 to 6 h to 10 to 15 min. Although there are costs involved to integrate and license automated tools, time savings could potentially quickly recoup any investment needed. Matching pharmacogenomic testing selection criteria to a specific site helps make patient selection more relevant for that location. In this quality improvement project, we demonstrated that, even within the gene panel that a site uses, medications from the site list may not contain all medications that have clinically actionable drug gene interactions according to current guidelines, as new evidence is published regularly. These additional medications, in combination with phenotype probability calculations, significantly changed the makeup of the patient group selected for testing.

This study was limited to investigating the differences in how two patient selection methods ranked a single patient population. Testing results were not available to determine the number of actual drug–gene interactions found by pharmacogenomic testing. Future studies examining testing results will help to inform the scenarios in which one method may be preferable for patient selection.

A limitation of our study was the use of retrospective claims data instead of medication lists reconciled by a pharmacist. It is possible that certain patients who might have benefited from testing were missed if they did not submit all medication claims to the insurance carrier used in the study. However, the use of retrospective claims data did allow for cost information that would have otherwise been lacking. Another limitation of our study is the available North American phenotype prevalence data. This data is unfortunately often heterogenous in terms of ethnicity. We anticipate results from in process large-scale research studies will help improve this limitation.

A formal time study was not conducted as a part of this project. However, in general, we predict that the automated method is faster, especially in large patient populations, as the work is done programmatically. We also did not conduct error testing, which is another potential pitfall for non-validated manual methods.

Our findings have several practical applications. We showed an efficient method to select patients most likely to benefit from pharmacogenomic testing with clinical actionable information directly applicable to their current care and medication list. Our selection method differs from methods commonly used to choose pharmacogenomic testing candidates (i.e., prescription count, ER costs, and drug interaction counts). We showed the feasibility of using site-customizable algorithms to tailor an automated approach to a local site. Future research is warranted to evaluate the post-test benefits of each selection method for both the patients and the healthcare system.

## 5. Conclusions

It is possible to identify a cohort of patients who would likely benefit from pharmacogenomic testing. Automated methods reduce the number needed to test for actionable findings, but the method used to identify a specific cohort should be based on site-specific genotype availability as well as program goals. The optimal approach for choosing which patients should undergo pharmacogenomic testing is an ongoing discussion. Health systems and insurers must balance testing patients currently taking multiple pharmacogenomic medications with the longer-term value of predictive testing, which allows better future medication decisions. Staying current with new and updated CPIC guidelines is challenging for sites who build their own alerts and testing panels as pharmacy resources are always limited. Automating tasks such as patient selection for pharmacogenomic testing, can assist in solving these challenges, freeing up clinician time to focus on providing clinical care.

## Figures and Tables

**Table 1:** Genes and medications included in the patient selection algorithms.

A. Gene	B. Medication Class	C. Both Methods	D. Manual Only	E. Automated Only
*CYP2C19*	antifungal	voriconazole		
	antiplatelet			cilostazol
		clopidogrel		
	benzodiazepine			clobazam
	proton pump inhibitor	esomeprazole		
			dexlansoprazole	
		lansoprazole		
		omeprazole		
		pantoprazole		
			rabeprazole	
	SSRI	citalopram		
		escitalopram		
		sertraline		
	tricyclic antidepressant	amitriptyline		
		clomipramine		
		doxepin		
		imipramine		
			trimipramine	
*CYP2D6*	5-HT3 antagonist	ondansetron		
	alpha adrenergic blocker			tamsulosin
	alpha agonist			clonidine
	antiarrhythmic			flecainide
				mexiletine
				risperidone
	anticholinergic			benztropine
	antiestrogen	tamoxifen		
	antihistamine			meclizine
	antipsychotic			haloperidol
				pimozide
				propafenone
	anxiolytic			buspirone
	atypical antipsychotic	aripiprazole		
				brexpiprazole
	beta blocker			metoprolol
				nebivolol
				timolol
	CNS stimulant			lisdexamfetamine
				methamphetamine
	pain	codeine		
			hydrocodone	
			oxycodone	
		tramadol		
	phenothiazine			promethazine
	serotonin modulator			vortioxetine
	SNRI	atomoxetine		
		venlafaxine		
	SSRI	fluvoxamine		
		paroxetine		
	tricyclic antidepressant	amitriptyline		
		clomipramine		
		desipramine		
		doxepin		
		imipramine		
		nortriptyline		
			trimipramine	
*CYP2C9*	angiotensin receptor blocker			azilsartan
	anticoagulant		warfarin	
	antiepileptic	phenytoin		
	nonsteroidal anti-inflammatory			celecoxib
				mefenamic acid
	sulfonylurea			glimepiride
*CYP3A5*	immunosuppressant	tacrolimus		
*CYP4F2*	anticoagulant		warfarin	
*SLCO1B1*	statin	simvastatin		
*TPMT*	immunosuppressant	azathioprine		
	purine antagonist	mercaptopurine		
	purine analog	thioguanine		
*VKORC1*	anticoagulant		warfarin	

Abbreviations: CNS, central nervous system; SNRI, serotonin-norepinephrine reuptake inhibitor; SSRI, selective serotonin reuptake inhibitor. To compare the populations selected by the algorithms, two-sample *t*-tests were used to test for differences in the following parameters: unique and average medication claim counts, drug interaction count and severity, ER visit count, ER claim costs, inpatient visit count, and inpatient claim costs.

**Table 2:** Patient demographics.

	Eligible Patients (*n* = 6916)	Manual Cohort (*n* = 218)	Automated Cohort (*n* = 286)	*p*-Value		
				Manual vs. Eligible Cohort	Automated vs. Eligible Cohort	Manual vs. Automated
Age, years (mean, SD)	35 ± 17.4	33 ± 16.8	34 ± 17.5	0.09	0.31	0.57
Sex (*n*, %)				0.07	0.03	0.95
Male	2647, 38%	97, 45%	128, 45%			
Female	4269, 62%	121, 55%	158, 55%			

**Table 3:** Comparison of selected cohorts.

Metric	Manual Cohort *n* = 218	Automated Cohort *n* = 286	*p* Value
Average unique medication count per patient, mean ± SD	9.8 ± 4.1	7.4 ± 3.9	<0.0001
Drug interactions mean (moderate-or-higher severity), mean ± SD	3.3 ± 2.6	1.1 ± 1.9	<0.0001
Drug interactions (moderate-or-higher severity), % patients	80.3%	40.2%	<0.0001
Average Pharmacogenetic Interaction Probability (PIP) score, % (range)	29% (0–81%)	62% (51–81%)	<0.0001
At least one ER Visit Q4 2018, % patients	26.6%	11.2%	<0.0001
ER visit claim cost, overall, mean ± SD	USD 558 ± 1150	USD 253 ± 873	0.001
Inpatient visit(s) Q4 2018, % patients	19.3%	6.6%	<0.0001
Inpatient claim cost, overall, mean ± SD	USD 4423 ± 16,981	USD 3607 ± 27,006	0.68

## Data Availability

Restrictions apply to the availability of these data. Data was obtained from GatorCare under strict data use agreement. The data are not publicly available due to the confidential nature of patient data.
